# Comparison of modular and nonmodular tapered fluted titanium stems in femoral revision hip arthroplasty: a minimum 6-year follow-up study

**DOI:** 10.1038/s41598-020-70626-6

**Published:** 2020-08-13

**Authors:** Shuo Feng, Yu Zhang, Yu-Hang Bao, Zhi Yang, Guo-Chun Zha, Xiang-Yang Chen

**Affiliations:** 1 Department of Orthopedic Surgery, Affiliated Hospital of Xuzhou Medical University, 99 Huaihai Road, Xuzhou, 221002 Jiangsu China; 2 Department of Orthopedic Surgery, Hyogo College of Medicine, 1-1 Mukogawacho, Nishinomiya, Hyogo Japan

**Keywords:** Anatomy, Diseases, Trauma

## Abstract

Both modular and nonmodular tapered fluted titanium stems are commonly used in revision total hip arthroplasty (THA). However, which type of femoral stem is superior remains controversial. The purpose of this study was to assess the clinical and radiographic outcomes of modular and nonmodular tapered fluted titanium. The clinical data of patients undergoing primary revision THA from January 2009 to January 2013 in two institutions were retrospectively analyzed. According to the type of prosthesis used on the femoral side, the patients were divided into the modular group (108 hips; Link MP modular stem in 73 hips and AK-MR modular stem in 35 hips) and nonmodular group (110 hips; Wagner SL stem in 78 hips and AK-SL stem in 32 hips). The operative time, hospital stay, blood loss, blood transfusion volume, hip function, hip pain, limb length discrepancy, imaging data, and complications were compared between the two groups.A total of 218 patients were followed up for 78–124 months, with an average of 101.5 months. The incidence of intraoperative fracture in the modular group (16.7%) was significantly higher than that in the nonmodular group (4.5%; (*P* < 0.05). At the last follow-up, the limb length difference in the modular group (2.3 ± 2.7 mm) was significantly lower than that in the nonmodular group (5.6 ± 3.5 mm; *P* < 0.05), and the postoperative prosthesis subsidence in the modular group (averaged 0.92 mm; 0–10.2 mm) was significantly less than that in the nonmodular group (averaged 2.20 mm; 0–14.7 mm; *P* < 0.05). Both modular and nonmodular tapered fluted titanium stems can achieve satisfactory mid-term clinical and imaging results in patients who underwent femoral revision. The modular stems have good control of lower limb length and low incidence of prosthesis subsidence.

## Introduction

Total hip arthroplasty (THA) is an effective treatment for end-stage hip disease. It not only relieves pain caused by hip disease but also improves hip function. In 2005, a total of 40,800 patients underwent hip revision surgery in the United States. These cases account for approximately 17.5% of all hip arthroplasty, and this number is expected to increase by 137% by 2030^1^. In the UK, the hip revision rate has exceeded 10% in 2015 and is expected to increase by 31% by 2030^2^. With the development of materials science, prosthesis design, and surgical techniques, the initial THA has been increased year by year around the world. Unfortunately, patients were found to be younger. Therefore, the revision surgery is also increasing yearly. In hip revision, 40% of patients require only acetabular revision, and 60% require femoral revision^3^.

The cement prosthesis will lead to a decrease in the cement–bone interface bonding strength. This reduction can affect the stability of the prosthesis, thereby influencing the long-term efficacy. Studies have shown that the cement–bone interface’s bonding strength is only 20.6% of the initial replacement. If the hip joint is subjected to re-revision, the intensity is only 6.8% of the initial replacement^3^. The loosening rate of cement prosthesis after revision is high^4,5^. In view of the high loosening rate after revision of the cemented prosthesis, some scholars proposed the use of a biological long-handle revision prosthesis of the femoral side in revision^6^. In North America, extensively coated cylindrical stems have been widely used for many years. However, concerns regarding severe postoperative thigh pain (8–9%), severe stress shielding of the proximal femur (6–7.6%), and high failure rate of patients with Paprosky type III femoral defects remain^7–9^. Many scholars^10^ believe that the tapered stem with ridge is more suitable when bone defects affect the rubbing requirements than cylindrical stems. During femoral revision, tapered stems can deal well with various bone defects.

Previous studies have shown that modular tapered fluted titanium stems have the advantages of easy adjustment of lower limb length, forward inclination, and eccentricity^11–13^. They also have some disadvantages such as high incidence of intraoperative fracture, corrosion, and fracture at the proximal and distal parts of the prosthesis^14,15^. Some scholars suggested the use of nonmodular tapered fluted titanium stems in femoral revision. They believe that the implantation of nonmodular tapered fluted titanium stems is simple, and the prosthesis does not have the disadvantages of corrosion and fracture. However, nonmodular tapered fluted titanium stems have the disadvantages of postoperative dislocation and high incidence of prosthesis sinking^16^.

At present, distal fixation femoral prostheses are mainly divided into modular and nonmodular tapered fluted titanium stems, and both types have been widely used. There is no consensus in the academic community on which prosthetic design is presently appropriate for femoral revision. However, in revision, there is no theoretical basis in choosing modular or nonmodular tapered fluted titanium stems. The criteria for clinical selection of modular or nonmodular tapered fluted titanium stems are often based on the preferences and experience of the operators. The selection of a suitable prosthesis can not only improve the success rate of revision surgery but also improve the prognosis of patients. Thus, the design characteristics, clinical efficacy, and radiographic results of the two kinds of prostheses should be compared to provide a basis for the clinical selection of revision prosthesis. Previous studies^17,18^ only explored the different early clinical effects of the two stems. The mid- and long-term efficacy of modular and nonmodular tapered fluted titanium remains uncertain. The purpose of this study was to assess the clinical and radiographic outcomes of modular and nonmodular tapered fluted titanium.

## Materials and Methods

### Patient selection

Patients who underwent revision THA with modular or nonmodular tapered fluted titanium stems in the Affiliated Hospital of Xuzhou Medical University and Hyogo College of Medicine Hospital from January 2009 to January 2013 were reviewed. This retrospective study was approved by the local Ethical Committee (Office for Research Ethics Committees Affiliated Hospital of Xuzhou Medical University). All methods were performed in accordance with the relevant guidelines and regulations. Informed consent was obtained from all patients.

A total of 239 patients (239 hips) were initially identified. Twelve patients were lost to follow-up, and 9 patients died of causes unrelated to their operation. The remaining 218 hips (218 patients) were analyzed. On the basis of the type of prosthesis used on the femur side, the patients were divided into the modular group (108 hips; Link MP modular stem for 73 hips and AK-MR modular stem for 35 hips) and nonmodular group (110 hips; Wagner SL stem for 78 hips and AK-SL stem for 32 hips). The general information of the patients in the two groups is shown in Table 1.

**Table 1:** Comparison of basic data between the two groups.

Classification	Modular group	Nonmodular group	*P* values
Age (years)	69.1 ± 7.5 (49–82)	67.6 ± 7.9 (50–83)	0.100
Gender (female/male)	48/60	50/60	0.136
BMI (kg/m^2^)	26.1 ± 2.8(19.15–32.30)	25.9 ± 2.5(20.74–31.99)	0.833
Initial replacement to repair time (months)	12.6 ± 6.0 (1–27)	11.0 ± 6.7 (0.08–25)	0.057
**Reasons for revision (n)**			0.583
Aseptic loosening	96	95	
Periprosthetic fractures	6	5	
Dislocation	6	10	
**Paprosky femoral defect (n)**			0.347
I	18	20	
II	54	60	
IIIA	24	25	
IIIB	12	5	
**ASA classification (n)**			0.168
I	6	10	
II	90	80	
III	12	20	
Combined acetabular revision(n)	96	105	0.071
VAS score (score)	7.6 ± 1.3 (6–10)	7.5 ± 1.1 (6–10)	0.839
Harris score (score)	40.5 ± 6.1 (29–52)	40.1 ± 6.6 (27–52)	0.774
Preoperative limb length discrepancy (mm)	18.7 ± 6.6 (5–33)	20.3 ± 6.1 (5–32)	0.071

There was no significant difference in gender, age, BMI, initial replacement to repair time, reasons for revision, type of bone defect, ASA classification, number of acetabular side revisions, preoperative Harris score, Visual Analogue Scale/Score (VAS) score, and limb length discrepancy (LLD) between the two groups.

### Clinical assessment

The patients were clinically evaluated on the basis of operation time, dominant blood loss (intraoperative blood loss + postoperative drainage volume), blood transfusion volume, hospitalization time, hip function, thigh pain, LLD, radiographic data (e.g., subsidence, bone growth, and osteolysis), and complications (intraoperative fracture, postoperative periprosthetic fracture, dislocation, infection, and heterotopic ossification). Hip function was assessed by the Harris hip score before surgery and during each visit. Thigh pain was assessed using the VAS scores^19^. LLD was assessed via a subjective measurement method^20^.

### Radiographic assessment

The degree of the femoral defect was evaluated from the preoperative X-ray according to the Paprosky classification^21^. Prosthetic subsidence was assessed by the criteria set forth by Callaghan et al.^22^. Stability of femoral prosthesis was assessed with the standard evaluation proposed by Engh et al.^23^. Heterotopic ossification was assessed using the Brooker^24^ standard.

### Statistical analysis

The analysis and production of data and charts were processed by IBM SPSS Statistics 19.0 statistical software (IBM, Chicago, IL, USA) and GraphPad Prism6.0 (GraphPad Software, San Diego, CA, USA). Continuous variables were analyzed using Wilcoxon rank-sum tests. Categorical variables were analyzed by the Pearson chi-square or Fisher exact tests. Kaplan–Meier survivorship analyses were conducted with the endpoint defined as any reoperation due to septic or aseptic complications and with the endpoint defined as any reoperation due to aseptic complications. Test level was set at both sides α = 0. 05, and *P* < 0.05 was considered statistically significant.

Power of the original study. The observational cohort study was powered to detect a distance of postoperative prosthetic subsidence as the minimum mean difference of significance, and the standardized difference (0.39) was calculated using the standard deviation (0.98) based on an earlier report by Huang et al.^17^. We estimated that 194 participants would be required to enable detection of significant difference at the 5% significance level with 85% power.

## Result

### Basic conditions of surgery

No significant differences were found between the two groups in terms of operation time, hospitalization time, blood loss, and blood transfusion (*P* > 0.05). A comparison of intraoperative data between the two groups is shown in Table 2.

**Table 2:** Comparison of intraoperative data between the two groups.

Classification	Modular group	Nonmodular group	P values
operative time (minutes)	235.4 ± 46.5 (120–330)	230.2 ± 61.2 (120–385)	0.188
hospital stay (days)	20.7 ± 4.4 (12–34)	20.4 ± 4.9 (10–40)	0.326
Intraoperative blood loss (ml)	1302.8 ± 326.8 (800–2100)	1232.3 ± 412.7 (300–2700)	0.059
Postoperative drainage (ml)	539.6 ± 91.4 (310–823)	522.2 ± 112.8 (315–774)	0.072
Total blood loss (ml)	1850.7 ± 345.3 (1240–2710)	1763.6 ± 450.6(680–3525)	0.067
Blood transfusion volume (ml)	785.2 ± 345.5 (400–1600)	712.7 ± 317.7 (400–1600)	0.125
Wire binding (n)	36	40	
Allograft bone plate(n)	18	10	
ETO (n)	12	15	

### Clinical results

A total of 218 patients were followed-up for an average of 101.5 months. The modular and nonmodular groups did not significantly differ in the most recent postoperative Harris hip score and VAS scores. The most recent postoperative Harris hip score increased from 40.5 ± 6.1 preoperatively to 86.4 ± 3.9 in the modular group (*P* < 0.05) and 40.1 ± 6.6 preoperatively to 85.5 ± 3.8 in the nonmodular group (*P* < 0.05). The final follow-up VAS score decreased from 7.6 ± 1.3 preoperatively to 1.9 ± 0.5 in the modular group (*P* < 0.05) and 7.5 ± 1.1 preoperatively to 1.8 ± 0.5 in the nonmodular group (*P* < 0.05). The leg length discrepancy of the modular group decreased from 18.7 ± 6.6 mm preoperatively to 2.3 ± 2.7 mm at the final follow-up (*P* < 0.05); the leg length discrepancy of the nonmodular group decreased from 20.3 ± 6.1 mm preoperatively to 5.6 ± 3.5 mm at the final follow-up (*P* < 0.05). At the final follow-up, the leg length discrepancy of the modular group was significantly lower than that of the nonmodular group (*P* < 0.05). A comparison of pain and hip function between the two group is shown in Table 3.

**Table 3:** Comparison of pain and hip function between the two groups.

Classification	Modular group	Nonmodular group	P values
Preoperative VAS score	7.6 ± 1.3 (6–10)	7.5 ± 1.1 (6–10)	0.839
Final VAS score	1.9 ± 0.5 (1–3)	1.8 ± 0.5 (1–3)	0.126
Preoperative Harris score	40.5 ± 6.1 (29–52)	0.1 ± 6.6 (27–52)	0.774
Most recent postoperative Harris Hip Score	86.4 ± 3.9 (78–96)	85.5 ± 3.8 (78–95)	0.085
Preoperative limb length discrepancy	18.7 ± 6.6 (5–33)	20.3 ± 6.1 (5–32)	0.071
Most recent postoperative limb length discrepancy	2.3 ± 2.7 (0–11)	5.6 ± 3.5 (0–15)	0.000

### Radiographic results

**Figure 1:**
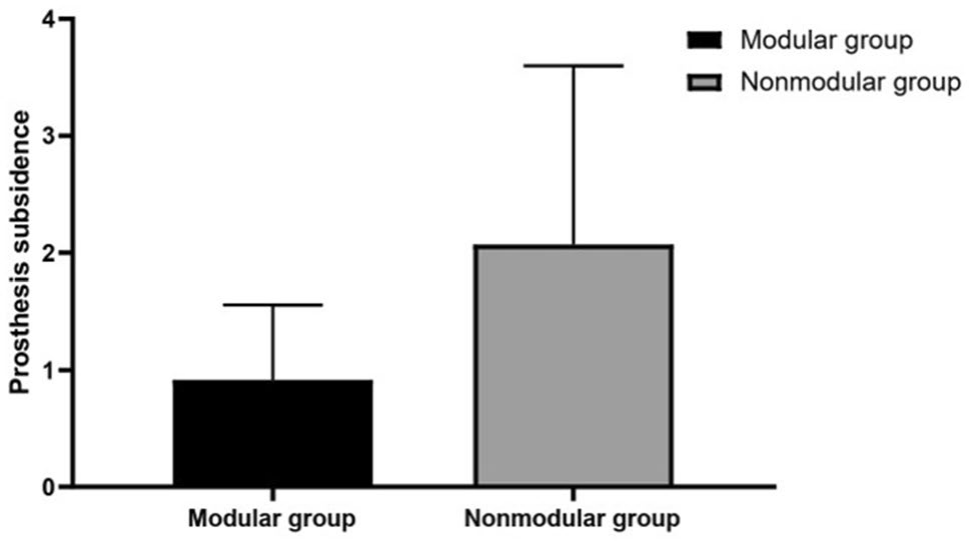
Comparison of the prosthesis subsidence between the two groups at the last follow-up (*P* < 0.05).

**Figure 2:**
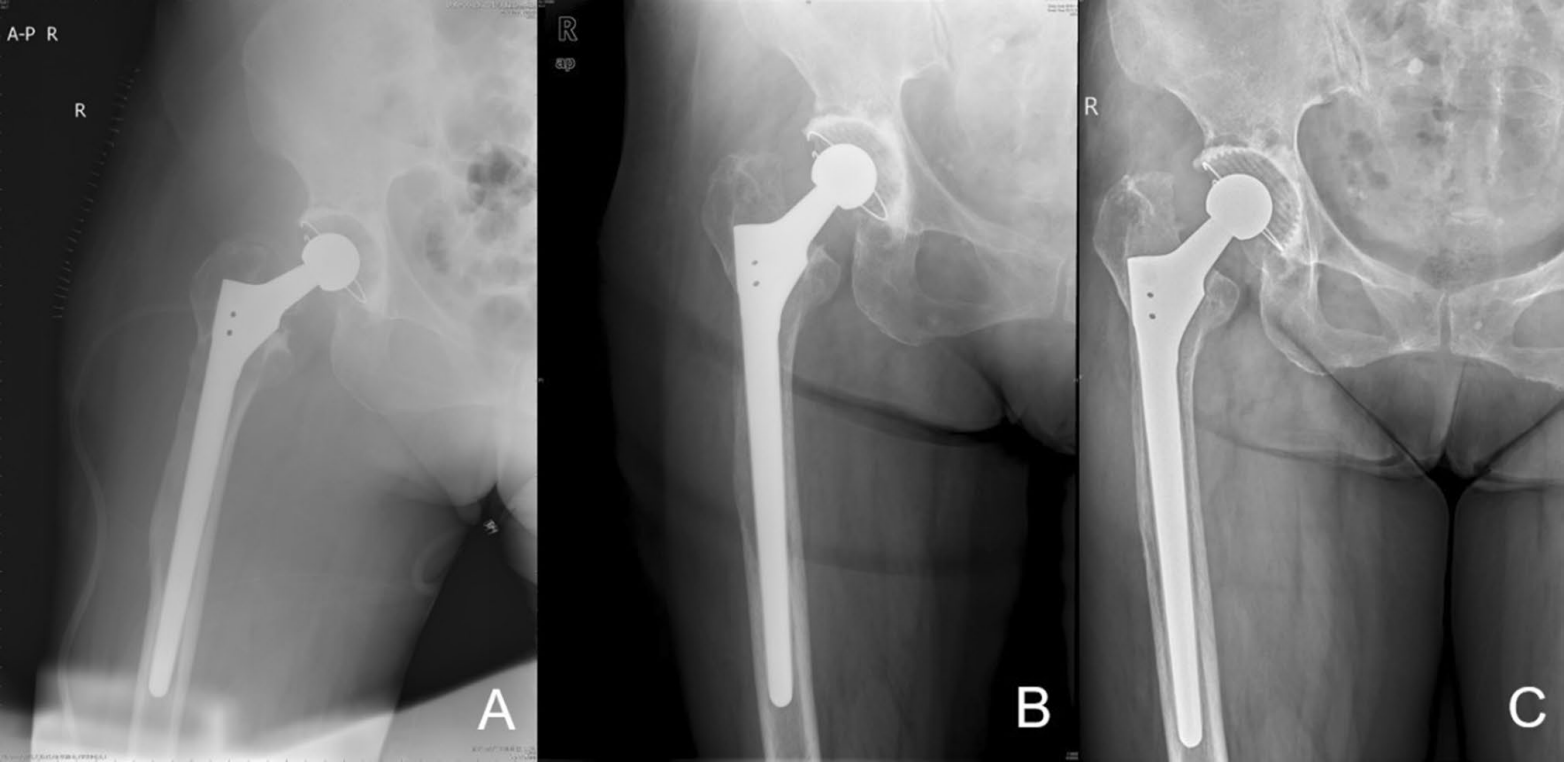
Postoperative radiographs of high-grade femoral defect managed with a nonmodular stem with stem subsidence.

**Figure 3:**
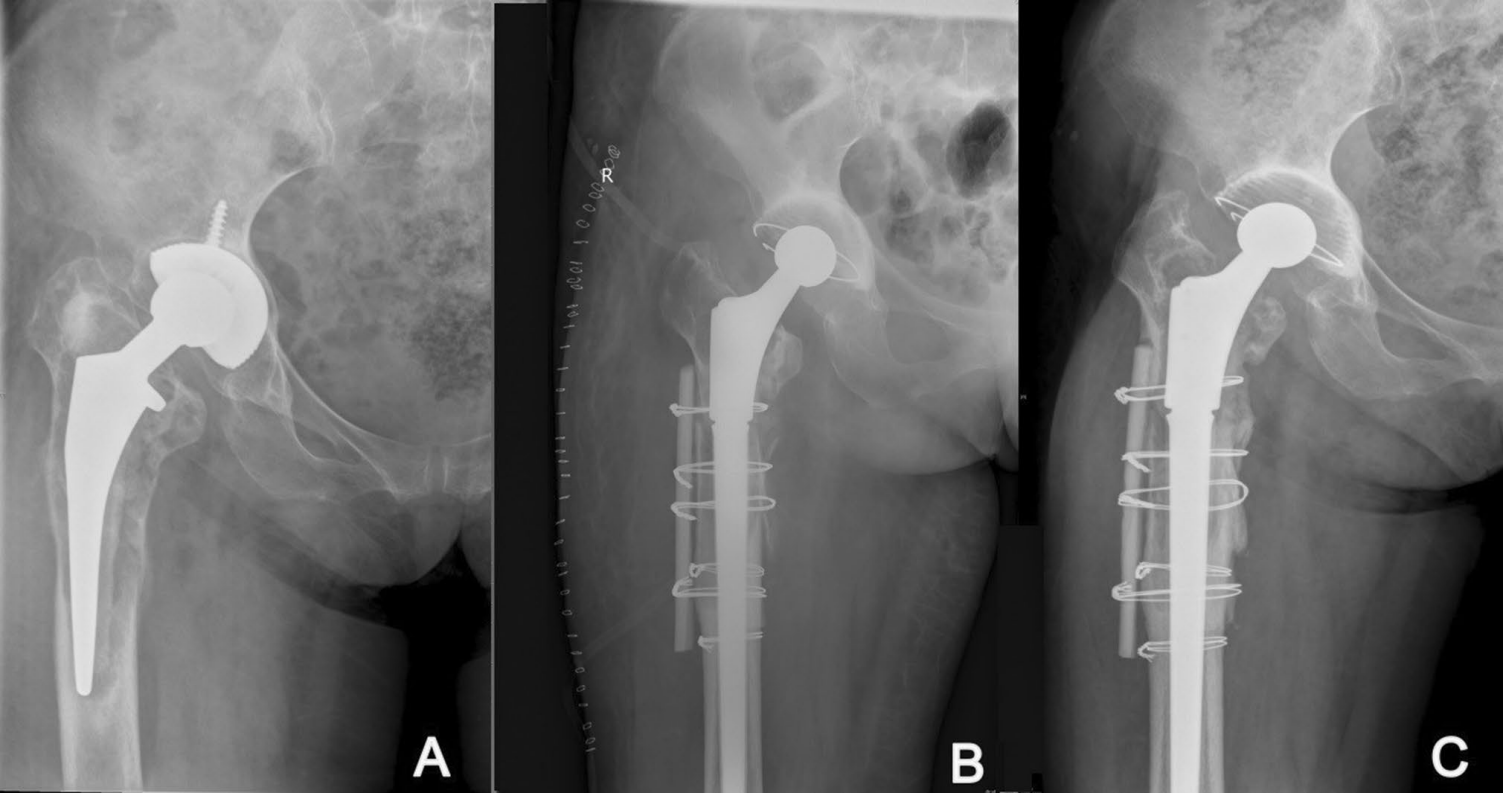
Preoperative and postoperative radiographs of high-grade femoral defect managed with a modular stem with failed osseointegration and stem subsidence.

At the final follow-up, the average subsidence in the modular group was 0.92 mm (range 0–10.2 mm), which was significantly lower than that in the nonmodular group (2.20 mm; range 0–14.7 mm; *P* < 0.05; Fig. 1). One patient in the modular group (1.0%) and three patients in the nonmodular group (2.7%) experienced subsidence greater than or equal to 10 mm (*P* = 1.0; Figs. 2 and 3). The patient in the modular group had a type IIIB femoral defect. By contrast, one patient in the nonmodular group had type IIIA femurs and two patients in the same group had a type IIIB femur. However, all subsided stems achieved secondary stability and osteointegration at the most recent follow-up. No dislocation caused by subsidence was found.

### Survivorship

**Figure 4:**
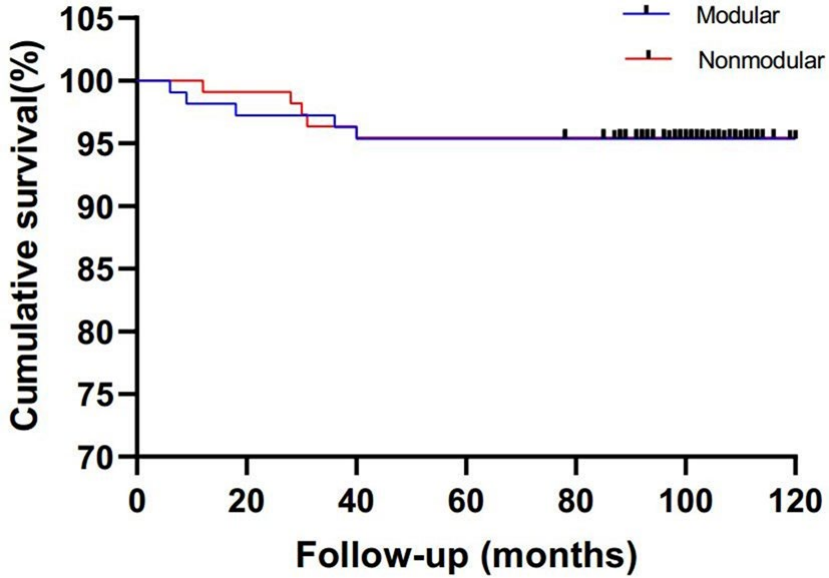
Kaplan–Meier survival analysis with the endpoint defined as any reoperation because of septic or aseptic complications.

**Figure 5:**
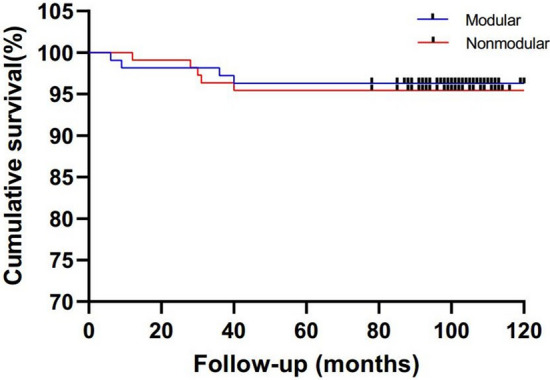
Kaplan–Meier survival analysis with the endpoint defined as any reoperation because of aseptic complications.

The 8-year cumulative survivorship with the endpoint defined as any reoperation due to septic/aseptic complications did not significantly differ between the modular (95.4%; 95% confidence interval [CI] 91.48–99.32%) and nonmodular groups (95.5%; 95% CI 91.58–99.42%; *P* = 0.969; Fig. 4). The 8-year cumulative survivorship with the endpoint defined as any reoperation due to aseptic complications did not significantly differ between the modular (96.3%; 95% CI 92.78–99.82%) and nonmodular groups (95.5%; 95% CI 91.58–99.42%; *P* = 0.759; Fig. 5). In addition, no group differences were found for the rates of reoperation due to aseptic complication or the total rates of reoperation due to septic and aseptic complications (Table 4).

**Table 4:** Comparison of reasons for reoperation between the two groups.

Reasons for reoperation	Modular group	Nonmodular group
Aseptic reasons	4	5
Periprosthetic fracture	2	2
Dislocation	0	3
Mechanical failure	2	0
Periprosthetic joint infection	1	0
Overall reason	5	5

### Postoperative complications

Intraoperative fractures occurred more frequently in the modular group (16.7%; 18 of 108) than in the nonmodular group (4.5%; 5 of 110; *P* < 0.05). Among the 18 cases of fracture in the modular group, femoral trochanteric fracture occurred in 12 cases (with wire banding), femoral shaft fracture was noted in six cases (with wire banding), and all fractures had bone healing. In the nonmodular group, only five cases suffered from femoral shaft fracture (with wire binding) and also demonstrated bone healing.

Two cases (1.9%) of periprosthetic fracture occurred in the modular group, and two cases (1.8%) of periprosthetic fracture were noted in the nonmodular group (*P* > 0.05). Patients with periprosthetic fractures were treated with open reduction and internal fixation.

No dislocation occurred in the modular group, and three cases (2.7%) of hip dislocation occurred in the nonmodular group (*P* > 0.05). Three cases of frequent dislocation after closed reduction was treated with replacement of lining and femoral head size.

Eighteen cases (16.7%) presented heterotopic ossification in the modular group (12 Brooker Degree I and 6 Degree II), whereas 20 cases (18.2%) demonstrated heterotopic ossification in the nonmodular group (16 Brooker Degree I and 4 Degree II). No significant differences were found in the two groups (*P* > 0.05).

No significant group differences were found in terms of rate of infection. The modular group only had one infected hip, whereas none was found in the nonmodular group. The patient in the modular group received multiple irrigations and debridements, with removal of the implant and revision with a new MP stem.

Two mechanical failures associated with the modular design were identified in the modular group. Both stems showed the locking screw backing out and disengaging, respectively, at 6 and 9 months postoperatively. The set screw was tightened without removal of the stem during reoperation (Table 5).

**Table 5:** Comparison of postoperative adverse events between the two groups.

Classification	Modular group	Nonmodular group	*P* values
Intraoperative fracture	18	5	0.004
Postoperative periprosthetic fractures	2	2	0.985
dislocation	0	3	0.251
Heterotopic ossification	18	20	0.768
infection	1	0	0.495
Mechanical failure	2	0	0.244

## Discussions

Consistent with previous studies^25,26^, the clinical efficacy, survivorship, and radiographic results were similar in femoral revision between the modular and nonmodular groups, indicating that both stem types obtained satisfactory results for femoral side revision. However, compared with the nonmodular group, the modular group exhibited a smaller LLD, lower prosthetic incidence, and higher incidence of intraoperative fracture.

Both modular (Link-MR and AK-MR) and nonmodular (Wagner SL and AK-SL) stems are grit-blasted prostheses with a conical groove design, which can increase the contact area with diaphyseal cortex and achieve axial stability with the tapered geometry. Eight longitudinal ribs ensure rotational stability. Both stems have a titanium shaft with a circular cross section and a 2° taper. Titanium alloy can significantly reduce the elastic modulus in both kinds of prostheses to reduce the incidence of postoperative stress shielding and thigh pain. The design difference between the modular and nonmodular prostheses is the distal end of the modular prosthesis, which has a 3° tilt angle that matches the shape of the femur. However, this design was not found in Wagner SL and AK-SL. In addition, the significant difference between modular and nonmodular prostheses is a proximal design. Wagner SL and AK-SL femoral prostheses have an integrated design, and the modular femoral prosthesis adopts a neck component design made of multiple components. A length-adjusting washer in the proximal segment allows the length of the stem to be extended by 30 mm. Surgeons can select different components depending on the actual situation to achieve the desired leg length and facilitate the adjustment of anteversion and eccentricity.

This study showed that the LLD of the modular group was significantly smaller than that of the nonmodular group. This difference was mainly because modular tapered fluted titanium stems can be used to precisely adjust the length of the lower limb through the combination of proximal components during the operation. However, due to the integrated design of nonmodular tapered fluted titanium stems, the limb length cannot be adjusted again after the prosthesis is inserted. Therefore, the length of the lower limb with modular tapered fluted titanium stems demonstrates better control than that with nonmodular tapered fluted titanium stems. When the biological prostheses are used in revision, the prostheses will exhibit different degrees of subsidence, and most of them occur in the first year after surgery. The intraoperative prosthesis and the femoral medullary cavity are insufficiently fitted, and sinking can occur when the lower limb is loaded with weight. In this study, the modular stem and nonmodular stem also experienced different degrees of postoperative subsidence. At the last follow-up, the postoperative subsidence in the modular tapered fluted titanium stems was significantly lower than that in the nonmodular tapered fluted titanium stems. Park et al.^27^ reported an average prosthetic subsidence of 1 mm in patients who underwent femoral revision with modular tapered fluted titanium stems. This finding was similar to the results of this study.

In this study, the average postoperative subsidence of prosthesis in the nonmodular group was 2.20 ± 1.94 mm. The subsidence rate was found to be greater than 10 mm in 4% and 24% of first-generation and second-generation Wagner SL stems, respectively^16,28,29^. In view of the high postoperative subsidence of the first- and second-generation Wagner SL stems, the modified third-generation Wagner SL stem is widely used in femoral revision. Although the postoperative subsidence is lower than the previous two generations, the subsidence was significantly greater compared with the modular tapered fluted titanium stems. The modular tapered fluted titanium stem is designed with a three-degree curvature to better match the femur, and the medullary cavity is better filled. The intraoperative prosthesis makes a good compression match with the femoral bone marrow cavity, while the nonmodular stem is designed without curvature. Therefore, an adequate match with the femoral bone marrow cavity may be difficult in nonmodular tapered fluted titanium stems. Second, surgeons need to consider the soft tissue tension of the hip joint when implanting nonmodular stems. Repeated tests are often performed to reduce both the risk of dislocation and the difficulty of reduction. Moreover, the distal part of the nonmodular stem is not fully fixed, and the immediate stability of the prosthesis cannot be guaranteed, which increases the risk of later prosthesis sinking or loosening. The distal part of the prosthesis can be firmly established when the modular stem is used. The femoral head and proximal component can be used to adjust the length, eccentricity, and forward inclination of the prosthesis, thereby reducing the length of limb discrepancy and the risk of prosthesis loosening and dislocation.

Concerns regarding subsidence related to the dislocation risk persist in the field. In our study, most of the reasons for dislocation were due to the poor place of the prosthetic position, leading to early postoperative dislocation; no dislocation caused by subsidence was found. Stem subsidence was early after weight bearing, but all prosthetic subsidence stopped within 1 year after operation. The patient’s soft tissue adapts to subsidence and maintains good tension. Moreover, the hip joint can be stably maintained to a certain extent due to scar contracture. Previous studies have shown that notable tapered stem subsidence after surgery is uncommon; the majority of stems become stable when it does occur, rarely going on to aseptic loosening^30,31^. Stem subsidence may have drawbacks, such as limb shortening and altered hip biomechanics^32,33^. Tangsataporn et al.^34^ reported 13 hips (13.1%) with subsidence of at least 10 mm. In that group, five of the 13 (38.5%) stems required repeat femoral revision because of stem aseptic loosening. Moreover, no dislocation caused by subsidence was found. In our study, dislocation did not occur in the modular group, and three cases (2.7%) of hip dislocation occurred in the nonmodular group. Park et al.^35^ believed that the low dislocation rate of the modular stem may be related to its ability to make appropriate adjustments to the length of lower limbs, eccentricity, soft tissue tension around the hip joint, and anterior angle with the help of the special design of proximal components during the revision.

In this study, 18 cases (16.7%) presented intraoperative fracture in the modular group. Huang^25^ reported an incidence of intraoperative fracture of modular tapered fluted titanium stems of 16.9%, and this percentage was similar to the results obtained in this study. Previous studies have reported that the fracture rate of modular tapered fluted titanium stems during femoral revision can reach 16–32%^36,37^, and patients with bone defects of Paprosky type IIIB–IV are likely to have fractures during surgery^25^. Pattyn et al.^37^ reported that the incidence of intraoperative fracture is as high as 32% when modular tapered fluted titanium stems are used for femoral revision, which may be correlated with the intraoperative operation. We speculate that the high incidence of intraoperative fracture of modular tapered fluted titanium stems may be related to the design of the modular prosthesis and the operation experience of the operator. Compared with periprosthetic fractures, dislocation is one of the more common complications in revision. In this study, three cases of dislocation in the nonmodular group were due to extreme hip flexion, adduction, and internal rotation; the patient was treated with replacement of the inner liner and the femoral head. The advantage of modular tapered fluted titanium stems is that the anterior inclination, eccentricity, and length of the lower limb can be adjusted by changing the neck component, thereby reducing the incidence of dislocation.

Some scholars believe that modular tapered fluted titanium stems are prone to fretting wear and corrosion fracture at the connection of proximal components. The proximal cervical junction is cylindrical and does not have a tapered design, allowing the stress concentration to increase. The proximal cervical component of the prosthesis is connected with the distal handle by a locking screw. If the locking screw fails, the teeth on both proximal and distal components will not bite each other, resulting in the proximal part to loosen, followed by mechanical separation. In the present study, the modular groups demonstrated two proximal segment dissociation, and the incidence of mechanical failure of the modular stem was similar to that in the study of Park et al.^27^. By contrast, the nonmodular tapered fluted titanium stems avoids the potential risk of modular fretting corrosion and joint dissociation.

The present study has several limitations to acknowledge. First, the retrospective nature of the study makes it prone to selection bias. Although prospective randomized controlled studies can better control confounders and selective bias, they are difficult to be carried out in revision surgery. Second, the sample size was limited, and the selection of prosthesis was limited. Therefore, the results obtained were limited and could not be widely generalized to all modular and nonmodular tapered fluted titanium stems. Given the low incidence of subsidence, this study is underpowered, limiting our ability to correlate subsidence with the risk of dislocation. A large investigation with more patients experiencing subsidence of the stem would be necessary to provide greater statistically significant information on the subsidence related to the dislocation risk for this finding. Third, the attending surgeons who performed the revision came from two different institutions.

In conclusion, both modular and nonmodular tapered fluted titanium stems can achieve satisfactory mid-term clinical and radiographic results in patients who undergo femoral revision. Furthermore, compared with nonmodular tapered fluted titanium stems, modular tapered fluted titanium stems have good control of lower limb length and low incidence of prosthetic subsidence.
